# Exosomes Derived from Runx2-Overexpressing BMSCs Enhance Cartilage Tissue Regeneration and Prevent Osteoarthritis of the Knee in a Rabbit Model

**DOI:** 10.1155/2022/6865041

**Published:** 2022-11-28

**Authors:** Jing Hu, Zheng-shuai Shi, Xiang-Zhong Liu, Han-tao Cai, Ao-fei Yang, Da-ming Sun, Liang-liang Xu, Yi Yang, Zhang-Hua Li

**Affiliations:** ^1^Wuhan Children's Hospital (Wuhan Maternal and Child Healthcare Hospital), Tongji Medical College, Huazhong University of Science & Technology, Wuhan 430015, China; ^2^Huanggang Hospital of TCM Affiliated to Hubei University of Chinese Medicine, Huanggang 438000, China; ^3^Department of Orthopedics, Wuhan Third Hospital, Tongren Hospital of Wuhan University, Wuhan 430060, China; ^4^Hubei University of Chinese Medicine, Wuhan 430061, China; ^5^Hubei Provincial Hospital of Traditional Chinese Medicine, Wuhan 430061, China; ^6^Wuhan Sports University, Wuhan 430079, China

## Abstract

**Objectives:**

Osteoarthritis is the leading disease of joints worldwide. Osteoarthritis may be treated by exosomes derived from Runx2-overexpressed bone marrow mesenchymal stem cells (R-BMSCs-Exos). R-BMSCs-Exos would promote the proliferation, migration, and phenotypic maintenance of articular chondrocytes.

**Methods:**

BMSCs were transfected with and without Runx2. Exosomes derived from BMSCs and R-BMSCs (BMSCs-Exos and R-BMSCs-Exos) were isolated and identified. Proliferation, migration, and phenotypic maintenance were determined *in vitro* and compared between groups. The mechanism for activation of Yes-associated protein (YAP) was investigated using small interfering RNA (siRNA). The exosomes' preventive role was determined *in vivo* using Masson trichrome and immunohistochemical staining.

**Results:**

R-BMSCs-Exos enhance the proliferation, migration, and phenotypic maintenance of articular chondrocytes based on the YAP being activated. R-BMSCs-Exos prevent knee osteoarthritis as studied *in vivo* through a rabbit model.

**Conclusions:**

Findings emphasize the efficacy of R-BMSCs-Exos in preventing osteoarthritis. Potential source of exosomes is sorted out for the advantages and shortcomings. The exosomes are then modified based on the molecular mechanisms to address their limitations. Such exosomes derived from modified cells have the role in future therapeutics.

## 1. Introduction

Osteoarthritis (OA) is the chronic joint disease world over [1, 2]. It affects around 10% men and 18% women of above 60 years age [3]. OA pathological characteristics include articular cartilage degeneration, secondary bone hyperplasia, and narrowing of joint space found on plain radiographs [4]. OA is clinically manifested through dysfunction, malformation, and pain [5]. OA is treated via the pharmacological, nonpharmacological, and surgical procedures [6, 7]. There is a symptomatic relief; however, the damaged cartilage tissue cannot be effectively repaired [8]. Timely prevention and reversal of OA progression are thus important.

Mesenchymal stem cells (MSCs) have the potential of repairing damaged tissues. MSCs can be isolated from tissues like synovium, perichondrium, adipose, muscle, periosteum, and bone marrow [9]. Bone marrow mesenchymal stem cells (BMSCs) were first isolated from bone marrow in 1968 [10]. Tissues through BMSCs specifically regenerate the cartilage [11]. The direct use of stem cells causes chromosomal variations and immunological rejection [12]. It is therefore important to efficiently use MSCs without risks.

The activation of resident cells via paracrine mechanism helps in cell-mediated tissue repairing [13]. Exosome-membrane-bound vesicles of 30-150 nm diameter are produced by all humor and cell types and are important for communication between the cells [14, 15]. Exosomes have the biological characteristics of the cells where they are derived from [16]. The direct use of such exosomes has no impact of chromosomal variation or immunogenicity [12]. There are limited studies available regarding OA therapy using exosomes derived from BMSCs (BMSCs-Exos) [17] while this work hypothesizes their effectiveness in preventing or reversing OA.

## 2. Materials and Methods

### 2.1. Ethics Statement

The experiments of animal model were approved by the Ethics Committee of Wuhan Third Hospital, Tongren Hospital of Wuhan University, and Guide for the Care and Use of Laboratory Animals was followed. These experiments were conducted in the Animal Management Center. The number and sufferings of included animals were kept minimal.

### 2.2. Isolation, Purification, and Characterization of rbBMSCs

BMSCs were isolated from bone marrow as the previous protocol [18]. The cells were induced to differentiate by converting to osteogenic, adipogenic, and chondrogenic differentiation mediums. Differentiation was analyzed by the cell surface markers using flow cytometer (CytoFLEX, USA). The details are given in Supplementary Method 1.

### 2.3. Adenovirus Transfection

Adenovirus Runx2 (Ad-Runx2) was transfected by using reported method [19]. The target gene Runx2 with 1015 bp-2559 bp in length was designed by Shanghai Jikai Biotech Co., Ltd and cloned into Teasy vector. Ad-Runx2 was stored at -80°C refrigerator (New Cell & Molecular Biotech, China). Manufacturer's protocol was followed to transfect Ad-Runx2 with GFP fluorescence into MSCs and observed the fluorescence intensity. Transfection efficiency was verified through western blot and qRT-PCR.

### 2.4. Isolation and Identification of Exosomes

Exosomes were isolated from the conditioned medium of BMSCs and Runx2-transfected BMSCs [20]. Nanoparticle tracking analysis (NTA) and transmission electron microscopy (TEM) determined the size and distribution of exosomes. Mark proteins (CD63, CD81, and TSG101) were extracted from exosomes using Total Exosome Protein Isolation Kit (Invitrogen, USA). Exosome uptake by chondrocytes was observed via the PKH-26-labeled exosomes. The details are provided in Supplementary Method 2.

### 2.5. RNA Isolation and qRT-PCR

Total RNA was extracted from the cells using TRIzol reagent (Invitrogen) and mRNA by RNeasy/miRNeasy Mini Kit (Qiagen). For mRNA, cDNA was synthesized using PrimeScript RT reagent Kit (Takara). qRT-PCR was performed on CFX96™ Real-Time System (Bio-Rad) using iTaq Universal One-Step RT-qPCR Kit (Bio-Rad). GAPDH was employed as internal reference for other mRNAs. The primer sequences are given in Supplementary Method 3. Experiments were performed in triplicate.

### 2.6. Protein Isolation and Western Blot

The adopted protocol was the same as our previous reports [21]. Total protein content was isolated from cell lysis buffer having EDTA, and equal amounts were loaded for western blotting. The antibodies of anticollagen type II (COL-II), anti-SOX9, Runx2, and anti-aggrecan primary antibodies were obtained from Abcam (Cambridge, MA, USA), anti-YAP, anti-CD63, anti-CD81, and anti-TSG101 from System Biosciences (Palo Alto, CA, USA) and anti-CTGF, anti-Ankrd1, and GAPDH from Protein Tech (Wuhan, China). COL-II was analyzed using 8% (wt/vol) SDS-PAGE. The experiments were conducted in triplicate.

### 2.7. *In Vitro* Response of Chondrocytes to BMSCs-Exos and R-BMSCs-Exos

#### 2.7.1. Isolation and Characterization of Articular Chondrocytes

Rabbit articular chondrocytes (rbACs) were isolated through sequential proteinase and collagenase digestions [22]. The rbACs of third passage 2 (P2) were seeded in 6-well plate as 10^5^ cells/cm^2^ prior to treatment for avoiding the phenotypic loss. The rbACs were stained and identified using toluidine blue, fluorescent acridine orange, and immunofluorescent type II collagen. The details are described in Supplementary Method 4.

#### 2.7.2. Proliferation of Chondrocytes

Chondrocytes with exosomes or Ad-Runx2 transfection were measured via the EdU Cell Proliferation Kit (Invitrogen, Beijing, China). Normal chondrocytes or chondrocytes transfected with exosomes of different ratios or empty vectors were seeded into 24-well plate for 12 h at the density of 2 × 10^4^ cells/well. The cell culture was added to EdU solution (ratio of 1000 : 1) and incubated for 2 h at room temperature, followed by washing with PBS, fixing by 4% paraformaldehyde for 30 min, and incubating in glycine solution for 8 min. Cultures were then digested using trypsin EDTA. Cells were washed with PBS having 0.5% Triton X-100, stained with Apollo solution, and incubated for 30 min in the dark. Finally, Hoechst 3334 solution was added into the cells, incubated for 20 min in the dark, and observed under fluorescent microscope.

Cell proliferation rate = Number of proliferative cells × 100%.

The experiment was performed in triplicate.

#### 2.7.3. Migration of Chondrocytes

Transwell assay determines the stimulating effect of BMSCs-Exos and R-BMSCs-Exos on chondrocytes. Around 5 × 10^4^ treated cells, present in 200 *μ*L medium depleted serum, were added to the apical chamber of 24-well 8 *μ*m pore-size transwell plate (Corning, Corning, NY, USA). 600 *μ*L of chondrocyte culture medium including exosomes was added to the lower chamber after 12 h of incubation at 37°C. The upper chamber of transwell plate was fixed with 4% PFA for 15 min, stained with 0.5% crystal violet for 10 min, and washed three times with PBS. A cotton swab was used to remove the cells which did not migrate to the lower surface. Five random fields per well were photographed under Leica microscope. The experiments were repeated three times.

### 2.8. Animal Studies

#### 2.8.1. Model Development and Groups

Twenty New Zealand male rabbits (body weight 2.5 ± 0.5 kg) were provided by Wuhan wan qian jia xing Biotech Co. Ltd (Lenience No. SCXK Hubei 2019-0011). Rabbits were raised in separate cages on normal diet. Animal laboratories were maintained with 12 : 12 hours light/dark cycles, temperatures of 23-25°C, and steady humidity of 55-70%. Based on Sasaki et al. [23], the rabbit knee was opened through medial parapatellar approach in sterile conditions and laterally dislocated the patella to expose the articular surface of femoral trochlea. OA model was developed by transecting the anterior cruciate ligament and medial meniscus. The wound was washed with saline and sutured. Inflammation was avoided by administering an intramuscular injection of 800 KU penicillin for 5 days. Rabbits were divided into 4 groups: [1] Con group (without surgery; received articular cavity injection of normal saline every time), 10 knee joints from 5 rabbits, and *n* = 10 (same for all groups); [2] Mo group (received articular cavity injection of normal saline on first day of every week from 2^nd^ to 6^th^ week after surgery); [3] BMSCs-Exos group (received articular cavity injection of 1 mL BMSCs-Exos transfect chondrocytes suspension (10 *μ*g exosomes/mL) every time); [4] R-BMSCs-Exos group (received articular cavity injection of 1 mL R-BMSCs-Exos transfect chondrocytes suspension (10 *μ*g exosomes/mL) every time). Anesthetic rabbits were sacrificed after eight weeks of surgery, and knee samples were recovered to evaluate the disease progression.

#### 2.8.2. ELISA Test

The knee joint synovial fluid cytokines IL-1*β* and TNF-*α* were detected by the ELISA kits from Biological Technology Company, Ltd., Nanjing, China. The detections were made at 450 nm on ultraviolet microplate reader (Thermo Scientific Corporation, MA, USA).

#### 2.8.3. Histology and Immunohistochemical Analysis

Knee cartilage samples were fixed in 4% paraformaldehyde. They were rinsed with pure water after 24 h and fixed in paraffin embedding. After series of treatments, the specimens were prepared as 2-3 *μ*m thick slices using frozen microtome. The tissue sections were processed by Masson trichrome staining. Collagen and cartilage were stained blue under the microscope. The modified O'Driscoll Histologic score assessed the degree of cartilage repair.

The fixed and embedded tissues were cut to 4 mm sections. They were treated with 3% H_2_O_2_ and sodium citrate. BMSCs were blocked and incubated overnight at 4°C with anti-COL-II, SOX9, and aggrecan antibodies and at 37°C for 1 h. After washing 3 times with PBS, the sections were added with secondary antibody solution, incubated at 37°C for 30 min, and washed 3 times with PBS. Sections were added with freshly prepared diaminobenzidine, counterstained with hematoxylin for 1 min, dehydrated, cleared, and mounted in neutral balsam. Positive control was set using antigen positive sections and NC by replacing the primary antibody with PBS. Pictures were taken, and counts made under an optical microscope.

Percentage of positive cells of COL − II, SOX9, and aggrecan in each field = Number of positive cells in each field/Total number of cells in each field.

### 2.9. Statistical Analysis

The data was expressed as mean ± standard deviation (SD). Cell countings adopted mixed model repeated measures (MMRM) and histological evaluations made by Kruskal-Wallis test. Data was statistically analyzed using SPSS v.23.0 (IBM SPSS, Chicago, IL, USA), GraphPad v.6.0 (San Diego, CA, USA), and Adobe Illustrator CS6 (Adobe, San Jose, CA, USA). *P* < 0.05 indicated the statistically significant difference.

## 3. Results

### 3.1. Characterizations of MSCs and Chondrocytes

**Figure 1:**
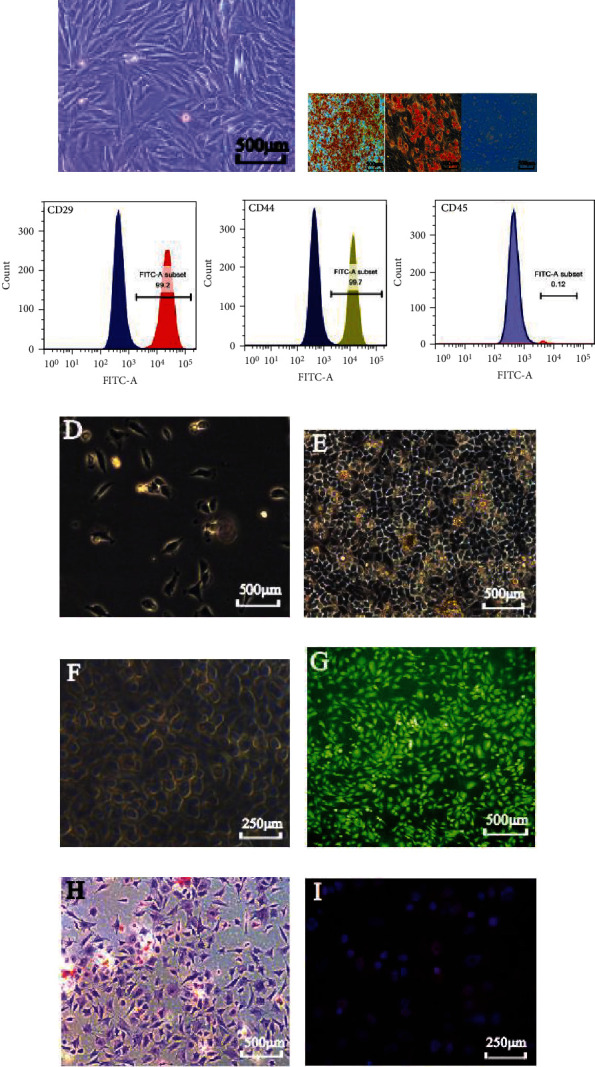
Characterizations of bone marrow mesenchymal stem cells (BMSCs) and chondrocytes, Original magnification, ×100. (a) BMSCs displayed a representative spindle-like morphology. (b) BMSCs exhibited multipotential differentiation for osteogenesis, adipogenesis, and chondrogenic. (c) BMSCs' surface markers proteins (CD29, CD44, and CD45) determined by flow cytometric. (d) Chondrocytes displayed a triangular or polygonal at the 3^rd^ day. (e) Chondrocytes grew and displayed a “paving stone” shape at the 7^rd^ day. (f) Chondrocytes matrix with refracted light was visible around the chondrocytes. (g–i) Chondrocytes was confirmed by staining of acridine orange fluorescence, toluidine blue, and immunofluorescence. This experiment was repeated independently three times.

Multiple cell colonies grow on the plate after culturing bone marrow for several days. Most passage 3 (P3) cells are long spindle-shaped (fusiform), and their clone growth is managed in a vortex (Figure 1(a)). P3 BMSCs proliferated in osteogenic, adipogenic, and chondrogenic media have differentiated into skeletal tissue (alizarin red: positive), adipose tissue (oil red O: positive), and cartilage tissue (alcian blue: positive) (Figure 1(b)). Flow cytometry identifies the surface markers of rbBMSCs. The cultured rbBMSCs meet the International Society for Cellular Therapy identification criteria of BMSCs [24], as cells are negative for CD45 (below 3%) and positive for CD44 and CD29 (above 95%) (Figure 1(c)). The isolated chondrocytes are triangular or polygonal on 3^rd^ day (Figure 1(d)) and “paving stone” shaped on 7^th^ day (Figure 1(e)). The chondrocytes matrix around chondrocytes are visible with refracted light (Figure 1(f)). Chondrocytes are confirmed by staining with fluorescent acridine orange (Figure 1(g)) and toluidine blue (Figure 1(h)). Type II collagen of chondrocytes representative proteins is evaluated by immunofluorescence staining (Figure 1(i)).

### 3.2. Ad-Runx2 Transfection Efficiency Test

**Figure 2:**
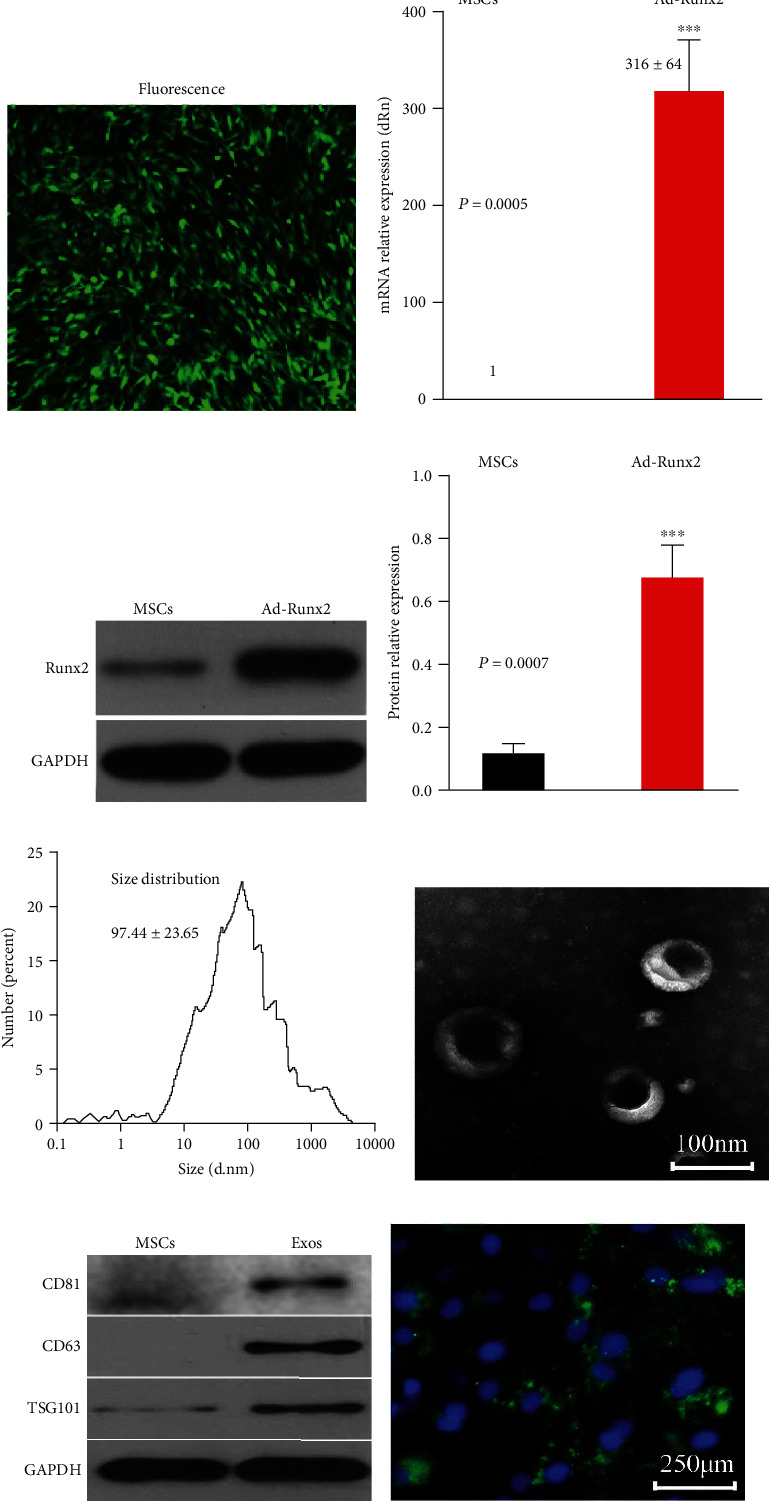
Ad-Runx2 transfection efficiency and exosome characteristics. (a) The green fluorescent distribution of BMSCs by microscope observation. Original magnification,×100. (b) The mRNA expression of Runx2 determined by qRT-PCR. (c, d) The protein expression of Runx2 measured by western blot analysis. (e) Particle size distribution of exosomes measured by nanoparticle tracking analysis (NTA). (f) Morphology of exosomes observed by transmission electron microscopy (TEM). (g) Exosome surface markers proteins (CD81, TSG101, and CD63) measured using western blotting. (h) Representative immunofluorescence photomicrograph of PKH-26- (green) labelled exosomes absorbed by chondrocytes, and the nuclei of which were stained by DAPI (blue). ^∗∗∗^*P* < 0.001 compared with MSCs, respectively, with the unpaired Student's *t*-test conducted. The statistical data were measurement data and expressed by means ± SE of 3 independent experiments. The experiment was performed in triplicates.

P3 BMSCs of transfection Ad-Runx2 show fluorescence with GFP green at 48 h and observed in fluorescent inverted microscope (Figure 2(a)). RNA is extracted for qRT-PCR detection, and transfection group increases (316 ± 64) (Figure 2(b)). Protein is extracted for western blotting, and band gray value is calculated. Transfection group is increased, and difference is significant (Figures 2(c) and 2(d)).

### 3.3. Isolation and Identification of Exosomes Derived from rbBMSCs

Nanoparticle tracking analysis (NTA), transmission electron microscopy (TEM), and western blotting are employed to characterize the particles secreted from BMSCs. Most particles range from 30 to 150 nm in size (Figure 2(e)). Particles display hollow spherical microvesicle morphology (Figure 2(f)). They are further analyzed by western blotting. The expressions of exosome markers CD63, CD81, and TSG101 (Figure 2(g)) are enriched in exosomes, and internalization occurs by articular chondrocytes. BMSCs are labelled using PKH-26 fluorescent dye before transfection with exosomes. The labelled exosomes are observed in perinuclear region of chondrocytes (Figure 2(h)) confirming the internalization by chondrocytes.

### 3.4. BMSCs-Exos and R-BMSCs-Exos Induced Proliferation, Migration, and Phenotypic Maintenance of rbACs

**Figure 3:**
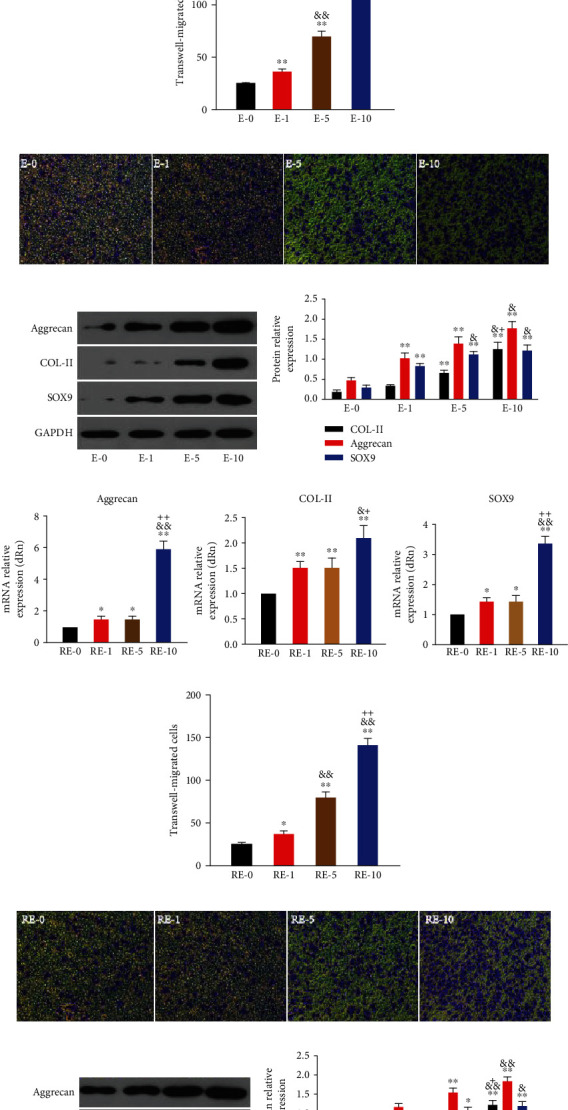
Responses of rbACs stimulated by BMSCs-Exos and R-BMSCs-Exos, and the 0, 1, 5, and 10 indicates that 0, 1, 5, or 10 *μ*g exosome concentrations/mL were used in the corresponding groups. (a) Gene expression changes of aggrecan, COL-II, and SOX9 after stimulation with different concentrations of BMSCs-Exos. (b, c) Cell migrated changes after stimulation with different concentrations of BMSCs-Exos. (d, e) Protein expression levels of aggrecan, COL-II, and SOX9 were detected by western blotting after stimulation with different concentrations of BMSCs-Exos, and the results of statistical analysis also shown. (f) Gene expression changes of aggrecan, COL-II, and SOX9 after stimulation with different concentrations of R-BMSCs-Exos. (g, h) Cell migrated changes after stimulation with different concentrations of R-BMSCs-Exos. (i, j) Protein expression levels of aggrecan, COL-II, and SOX9 were detected by western blotting after stimulation with different concentrations of R-BMSCs-Exos, and the results of statistical analysis also shown. This experiment was repeated three times. ^∗^*P* < 0.05 compared to 0; ^∗∗^*P* < 0.01 compared to 0; ^&^*P* < 0.05 compared to 1, ^&&^*P* < 0.01 compared to 1; ^+^*P* < 0.05 compared to 5; ^++^*P* < 0.01 compared to 5.

The exosomes functionality acts as regulatory signal for cargo transport [25]. The rbACs are incubated with BMSCs-Exos and R-BMSCs-Exos to determine their effect on articular chondrocytes. Chondrocytes are stimulated by 0, 1, 5, or 10 *μ*g/mL of BMSCs-Exos and R-BMSCs-Exos for 24 h. The qRT-PCR and western blotting assays are conducted. The levels of aggrecan, COL-II, and SOX9 genes are enhanced in dose-dependent manner (Figures 3(a) and 3(f)). R-BMSCs-Exos (Figure 3(f)) significantly promote articular chondrocytes-related gene expression compared with BMSCs-Exos (Figure 3(a)). The cell migrated ability measured by transwell assay. R-BMSCs-Exos (Figures 3(b) and 3(c)) significantly promote articular chondrocytes migrated compared with BMSCs-Exos (Figures 3(g) and 3(h)). Protein expression using western blotting shows same pattern. Compared with BMSCs-Exos (Figures 3(d) and 3(e)), aggrecan, COL-II, and SOX9 show higher protein expression levels for R-BMSCs-Exos (Figures 3(i) and 3(j)). BMSCs-Exos and R-BMSCs-Exos thus promote phenotypic maintenance.

**Figure 4:**
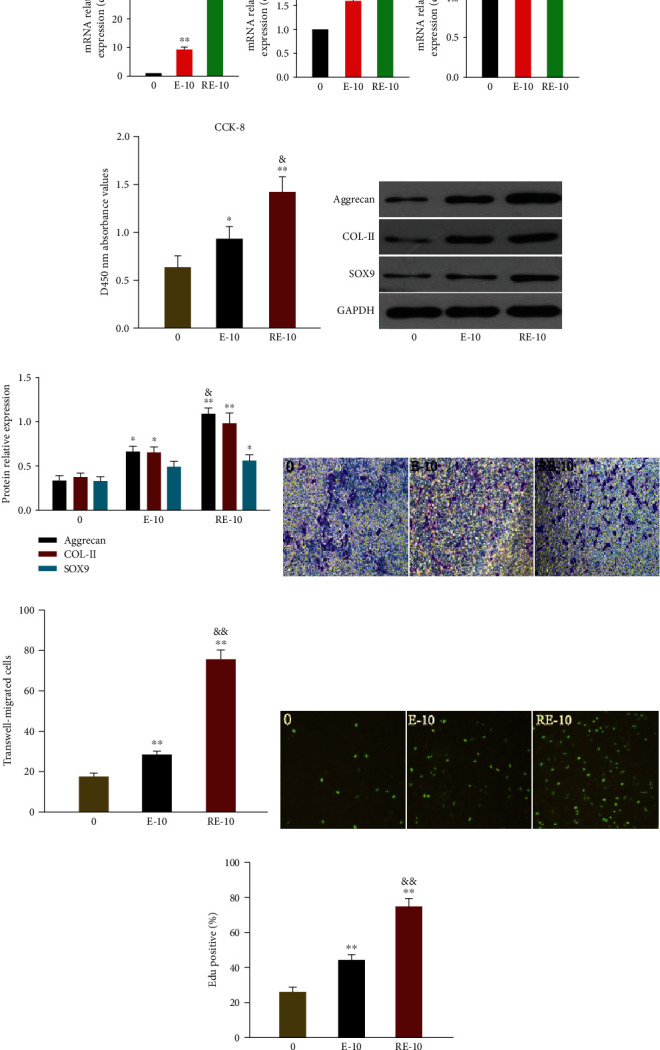
Responses of rbACs stimulated by BMSCs-Exos and R-BMSCs-Exos in 10 *μ*g exosome concentrations/mL. The E-10 indicates the BMSCs-Exos; the RE-10 indicates the R-BMSCs-Exos. (a) Gene expression changes of aggrecan, COL-II, and SOX9 after stimulation by E-10 and RE-10. (b) The OD value of rbACs measured by CCK-8 assay. (c, d) Protein expression levels of aggrecan, COL-II, and SOX9 were detected by western blotting after stimulation by E-10 and RE-10, and the results of statistical analysis also shown. (e) The number of invaded rbACs measured by transwell assay. Original magnification, ×200. (f) Quantitative analysis of the number of cell invasion. (g) Proliferation of rbACs determined by EdU labeling assay. Original magnification, ×200. (h) Quantitative analysis for EdU-positive cells. This experiment was repeated three times. ^∗^*P* < 0.05 compared to 0; ^∗∗^*P* < 0.01 compared to 0; ^&^*P* < 0.05 compared to E-10; ^&&^*P* < 0.01 compared to E-10.

Aggrecan, COL-II, and SOX9 genes are the markers of cartilage phenotype. Western blotting and qRT-PCR assays reveal that R-BMSCs-Exos promote more cartilage phenotypic maintenance compared with BMSCs-Exos (Figures 4(a), 4(c), and 4(d)). Proliferation ability is also evaluated by using CCK-8 and EdU. Compared with BMSCs-Exos, R-BMSCs-Exos bring more increase in chondrocytes proliferation (Figures 4(b), 4(g), and 4(h)). Transwell assay demonstrates higher number of invasive cells in R-BMSCs-Exos than in BMSCs-Exos (Figures 4(e) and 4(f)). R-BMSCs-Exos have thus the better potential in promoting the proliferation, migration, and phenotypic maintenance of rbACs.

### 3.5. BMSCs-Exos Induced Proliferation, Migration, and Phenotypic Maintenance of rbACs through YAP Activation

**Figure 5:**
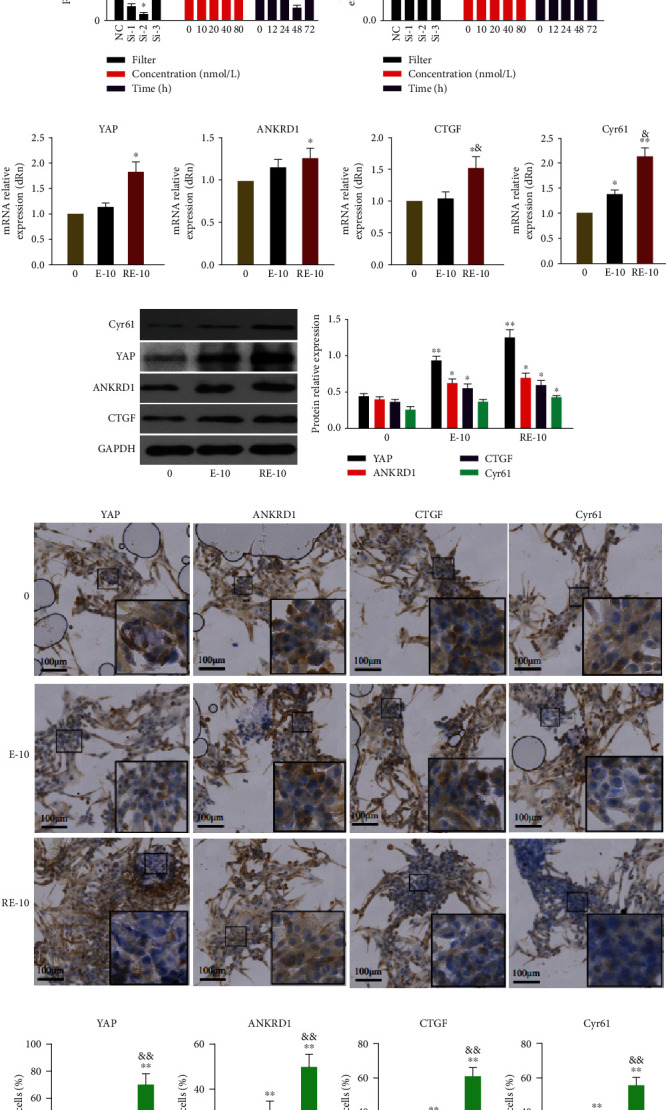
Filtering and function of YAP-siRNA. (a, b) YAP-siRNA protein expression levels of filter, action concentration, and action time were detected by western blotting and the results of statistical analysis. ^∗^*P* < 0.05 compared to NC or 0; ^∗∗^*P* < 0.01 compared to NC or 0. (c) The expression of filter, action concentration, and action time determined by qRT-PCR. ^∗^*P* < 0.05 compared to NC or 0; ^∗∗^*P* < 0.01 compared to 0. (d) Gene expression changes of YAP and its target genes (ANKRD1, CTGF, and Cyr61) were detected by qRT-PCR. ^∗^*P* < 0.05; ^∗∗^*P* < 0.01 compared to 0; ^&^*P* < 0.05 compared to E-10. (e, f) Protein expression levels of YAP and target genes including ANKRD1, CTGF, and Cyr61 were detected by western blotting, and the results of statistical analysis also shown. ^∗^*P* < 0.05 compared to 0; ^∗∗^*P* < 0.01 compared to 0. (g, h)Gene expression changes of YAP and its target genes (ANKRD1, CTGF, and Cyr61) were detected by immunohistochemical staining.^∗∗^*P* < 0.01 compared to 0, ^&&^*P* < 0.01 compared to E-10. This experiment was repeated three times.

The expression levels of YAP and target genes including ANKRD1, CTGF, and Cyr61 are analyzed to study the mechanism in chondrocytes stimulated by BMSCs-Exos. Three sequences of YAP-siRNA are filtered by western blotting and qRT-PCR where the inhibitory effect of si-2 is prominent. The dose-effect of YAP-siRNA-2 is analyzed, and the inhibitory effect of 40 nmol/L for 48 h is the strongest (Figures 5(a)–5(c)). It is also studied if the change in chondrocyte function is caused by YAP activation. Moreover, whether YAP is a key gene that makes BMSCs-Exos to stimulate chondrocytes for its functioning, YAP, ANKRD1, CTGF, and Cyr61 genes are detected by western blotting, RT-qPCR, and immunohistochemistry staining where their levels are enhanced (Figures 5(d)–5(h)).

**Figure 6:**
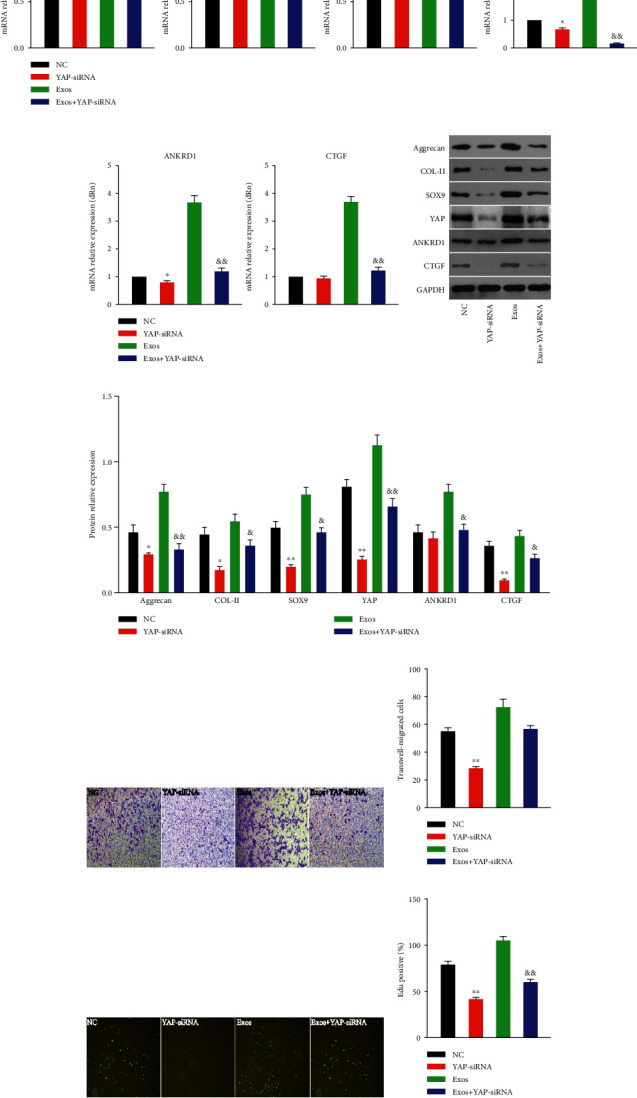
YAP plays an important role in mediating the effects of BMSCs-Exos stimulation. (a, b) Gene expression changes of aggrecan, COL-II, SOX9, YAP, ANKRD1, and CTGF after silence by YAP. The mark on the horizontal line applies to Figure 6. (c, d) Protein expression levels of aggrecan, COL-II, SOX9, YAP, ANKRD1, and CTGF were detected by western blotting after silence by YAP, and the results of statistical analysis also shown. (e, f) The number of invaded rbACs measured by transwell assay, and the results of statistical analysis also shown. Original magnification, ×200. (g) Proliferation of rbACs determined by EdU labeling assay. Original magnification, ×200. (h) Quantitative analysis for EdU-positive cells. This experiment was repeated three times. ^∗^*P* < 0.05; ^∗∗^*P* < 0.01 compared to NC; ^&^*P* < 0.05; ^&&^*P* < 0.01 compared to Exos.

YAP gene is silenced using small interfering RNA to verify the YAP role. Exos + YAP-siRNA group aggrecan, COL-II, SOX9, YAP, ANKRD1, and CTGF genes, and proteins are reduced compared to Exos (Figures 6(a)–6(d)). The proliferation and migration abilities are restricted in chondrocytes transfected with YAP-siRNA and cannot be enhanced by BMSCs-Exos (Figures 6(e)–6(h)).

### 3.6. R-BMSCs-Exos Preventing OA

**Figure 7:**
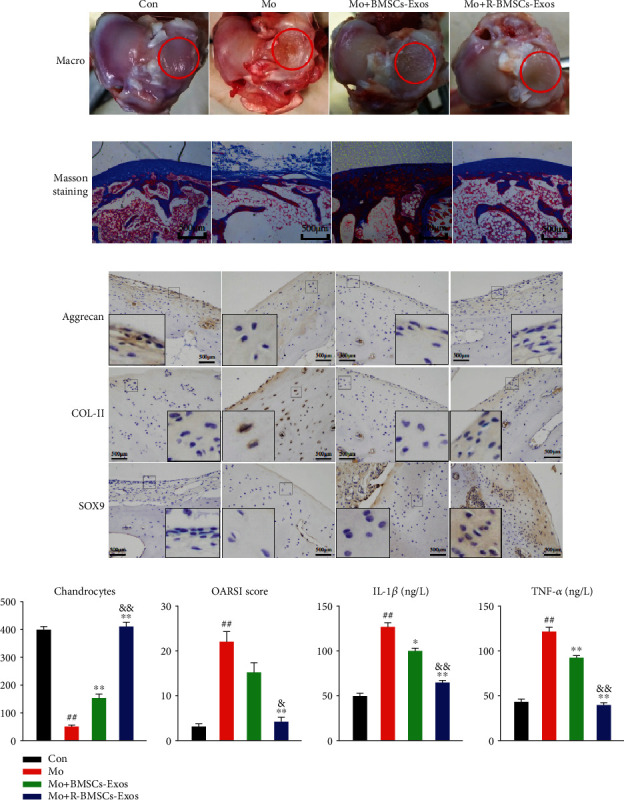
R-BMSCs-Exos prevented OA. (a) Observe the cartilage plane of the knee joint through the eyes. (b) Sections of femoral condyle were stained using Masson trichrome staining (Scale bar: 500 *μ*m). (c) Photomicrographs of femoral condyle sections stained using anti-type II collagen, anti-SOX9 or anti-aggrecan as primary antibodies (Scale bar: 500 *μ*m). (d) Statistical results of chondrocytes counted in randomly-selected high magnification fields and the result of statistical analysis of OARSI score in each group; IL-1*β* and TNF-*α* value in the joint fluid. This experiment was repeated three times. ^##^*P* < 0.01 compared to Con; ^∗^*P* < 0.05, ^∗∗^*P* < 0.01 compared to Mo;^&^*P* < 0.05, ^&&^*P* < 0.01 compared to Mo + BMSCs-Exos.

The potential of exosomes for OA prevention is verified in OA rabbit model (Figure 7). No obvious adversity is observed in each experimental group. In Mo group, severe joint wear and cartilage matrix loss are recorded. The expressions of COL-II, aggrecan, and SOX9 in cartilage have decreased while high expressions of IL-1*β* and TNF-*α* in joint fluid are observed. In Mo + BMSCs-Exos group, joint wear and cartilage matrix loss are also found; however, the impact is less severe compared to Mo group. The cartilage matrix consisting of COL-II is thin, and chondrocytes are arranged in dense clusters of cartilage cells rather than in the normal arrangement of neat rows. Expressions of aggrecan and SOX9 are high while those of IL-1*β* and TNF-*α* are low. In Mo + R-BMSCs-Exos group, the joint wear is mild. The cartilage matrix consisting of COL-II is slightly thinner than in the Con group but better than in Mo or Mo + BMSCs-Exos groups. There is an increase of aggrecan, COL-II, and SOX9 expressions compared with Mo group. No obvious TNF-*α* expression difference is observed in the joint fluid compared with Con.

The results implicate that R-BMSCs-Exos slow the progression of early OA and prevent the severe trauma to knee articular cartilage. The chondrocyte counts and OARSI scores are shown in Figure 7(d).

### 3.7. Summary of Therapeutic Mechanism

**Figure 8:**
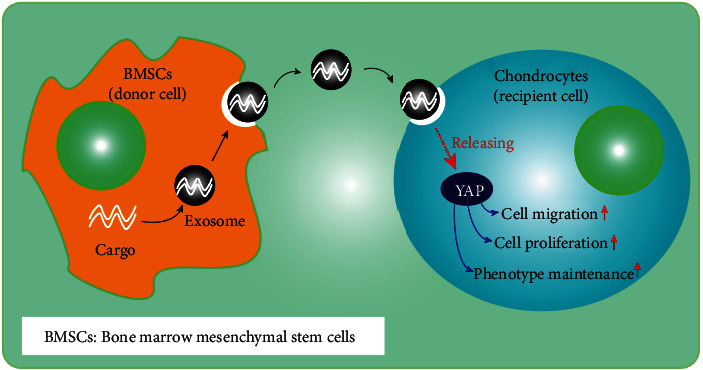
Diagram illustrating the proposed mechanism of action of BMSCs-Exos in OA.

The action mechanism of BMSCs-Exos is summarized in Figure 8. BMSCs-Exos activate the YAP signal pathway. YAP activation increases the chondrocyte proliferation, migration, and phenotype maintenance with decrease in IL-1*β* and TNF-*α* expressions. Runx2 expressed in BMSCs-Exos brings chondrocyte proliferation, migration, phenotype maintenance, and inflammatory indicators close to normal.

## 4. Discussion

Articular cartilage has limited potential of self-regeneration due to the lack of potential stem cell niches and cartilage precursor cells [26]. Runx2 overexpression in BMSCs promotes cartilage regeneration in rabbit knee articular cartilage defect model [21]. The overexpression of Runx2 adenovirus has some side effects, and its action mechanism is not clear. R-BMSCs-Exos avoid these shortcomings.

BMSCs are isolated from bone marrow which is an ideal source of seed cells due to easy availability and high differentiation potential [27–29]. Tao et al. found that synovial mesenchymal stem cells (SMSCs) can maintain their multidirectional differentiation potential after 10^th^ generation [30]. Mesenchymal stem cells (MSCs) family can thus be used for tissue regeneration [31, 32]. MSCs are a treatment for cartilage tissue damage [33].

Previous studies show that paracrine mechanisms including exosomes are responsible for stem cell or progenitor cell-mediated tissue regeneration [13, 34]. Exosomes derived from BMSCs (BMSCs-Exos) promote chondrocyte proliferation, migration, and phenotype maintenance; however, they have no specific pathway. YAP is stimulated at normal and silent levels where YAP and its target genes change accordingly. YAP promotes articular chondrocyte proliferation, migration, and phenotype maintenance and decreases inflammatory indicators in knee joint fluid.

YAP is responsible for signal transduction from cell membrane to nucleus and promotes cell proliferation [35, 36]. The precise mechanism of YAP in chondrocyte differentiation is indistinct. Early chondrocyte proliferation is promoted, and maturation is inhibited [37]. This phenomenon and the use of BMSCs-Exos to activate YAP produce similar results. The inhibition of SOX9 and its downstream genes (aggrecan and type II collagen) is the key to YAP-induced chondrocyte maturation inhibition. SOX9 shows good therapeutic potential.

As we all know, Runx2 has a direct effect on cartilage, so we did not pay attention to it at first, but we discovered through high-throughput sequencing that some genes that are beneficial to cartilage regeneration, such as miR-92a-3p, are highly expressed in R-BMSCs-Exos [38, 39]. Previous studies demonstrate that Runx2 plays important role in the regulation of chondrocyte differentiation and matrix degradation [40]. Runx2 is a positive regulator of chondrocyte differentiation and vascular invasion. Runx2 may promote chondrogenesis by maintaining or by initiating early chondrocyte differentiation [41]. Runx2 promotes cartilage repair and maintains the cartilage phenotype in knee articular cartilage defect [21]. Nucleic acids can be incorporated into exosomes through elevated intracellular RNA concentrations obtained by the overexpressed nucleic acids using adenovirus-based or lipid-based systems. Runx2 is overexpressed in BMSCs where Runx2 is enriched in its derived exosomes (R-BMSCs-Exos). Runx2-overexpressed BMSCs are the superior cell lines for preventing OA.

OA rabbit model is developed based on the knee joint instability induced by surgery. This model can simulate the knee joint instability caused by knee injury including the anterior cruciate ligament injury. It can also simulate joint wear caused by anatomical factors. R-BMSCs-Exos are used in OA rabbit model. The procession of early stage OA is delayed, and knee joint cartilage damage caused by OA is prevented by R-BMSCs-Exos, whereas the effect of BMSCs-Exos is limited.

There are still several questions that need to be addressed. The future studies may include the impact of concentration and duration of action of BMSCs-Exos. The rabbit model is chosen for this work; however, mouse model being closer to humans may be selected. These attempts will diversify the findings.

## 5. Conclusions

Combined data analyses from this study reveal that BMSCs and their exosomes with or without a type of modification show potential for future study and their use in clinical practice.

## Data Availability

The data used to support the findings of this study are available from the corresponding author upon request.

## Abbreviations

BMSCs-Exos: Exosomes derived from bone marrow mesenchymal stem cells
R-BMSCs-Exos: Exosomes derived from Runx2 overexpression bone marrow mesenchymal stem cells
OA: Osteoarthritis
rbACs: Rabbit articular chondrocytes
Ad-Runx2: Runx2 adenovirus
SMSCs: Synovial mesenchymal stem cells
qRT-PCR: Quantitative real-time polymerase chain reaction
PBS: Phosphate buffered saline.

## Supplementary Materials

Supplementary Materials Supplementary Method 1: isolation and identification of BMSCs. Supplementary Method 2: isolation and identification of exosomes from BMSCs. Supplementary Method 3: RT-qPCR gene sequence. Supplementary Method 4: isolation and identification of chondrocytes.
