# Supplementary material for: Exosomes Derived from Runx2-Overexpressing BMSCs Enhance Cartilage Tissue Regeneration and Prevent Osteoarthritis of the Knee in a Rabbit Model
Source: Stem Cells Int. 2022 Nov 28;2022:6865041. doi: 10.1155/2022/6865041 (PMC11401735; doi:10.1155/2022/6865041)
Supplement: Supplementary Materials — Supplementary Method 1: isolation and identification of BMSCs. Supplementary Method 2: isolation and identification of exosomes from BMSCs. Supplementary Method 3: RT-qPCR gene sequence. Supplementary Method 4: isolation and identification of chondrocytes. [file 6865041.f1.docx]

**Supplementary Methods**

1. **Isolation, purification and characterization of rbBM-MSCs**

**Isolation and purification of rbBM-MSCs**

New Zealand rabbits (body weight 2±0.5 kg) provided by the Wuhan wan qian jia xing Biotech Co., Ltd (Lenience No. SCXK Hubei 2016-0011). Heparin anti-coagulated bone marrow was collected from the rabbit proximal tibia under sterile conditions. Bone marrow were washed with washing buffer and centrifuged at 1500 rpm for 5 min. The cell pellet was resuspended in Dulbecco’s modified Eagle’s medium. Cell cultures were maintained in a 37˚C incubator with 5% CO_2_ and passaged. MSCs were purified and amplified (MSCs complete medium, ScienCell, USA). Some of the primary cultured cells were preserved at -80°C using CELLSAVING (New Cell & Molecular Biotech, China). The remainder were cultured and passaged for follow-up study.

**Multiple differentiation potential**

At passage 3 (P3), the cells were induced to differentiate by converting to osteogenic and adipogenic differentiation medium. Osteogenic differentiation was induced by replacing the complete culture medium with osteogenic medium (Cyagen, China) and culturing for 2 weeks. For adipogenic differentiation, the StemPro Adipogenesis Differentiation Kit (Gibco, USA) was used for 2 weeks. The procedure of inducing differentiation was as described in the manufacturer's instruction.alizarin red and oil red O staining kit (Cyagen Biosciences, USA) according to the manufacturer's instructions experimenting.

**Surface markers of rbBMSCs**

The phenotypic characterization was done by flow cytometry (CytoFLEX, USA). For his purpose, rbBMSCs from passage 3 were trypsinized and approximately 106 cells were washed twice with PBS containing 3% FBS, and were incubated with fluorescein isothiocyanate (FITC)-conjugated anti-CD29 (ab255354)、anti-CD44 (ab189524) and anti-CD45 (ab40763) (all from Abcam, USA) in washing buffer for 30 min on ice. rbBMSCs were washed with washing buffer again and FACSCalibur system (BD Biosciences, San Diego, CA) was used for characterization of rbBMSCs. Data were analyzed using with the FlowJo software (version 6.2).

1. **Isolation and identification of exosomes**

**Exosomes isolation from BMSCs**

When BMSCs reached 50-60% confluence they were washed with PBS, then replaced with MSCs medium containing 10% exosome-Depleted Fetal Bovine Serum (C38010050, Shanghai XP Biomed Ltd, China) for an additional 48h at 37°C in an atmosphere of 5% humidified CO_2_. The conditioned medium was collected and centrifuged at 1000× g for 10 min at 4°C and then at 2,500 × g for 15 min to remove dead cells. After centrifugation, the supernatant was filtered using a 0.22 μm filter (PR03683, Merck Millipore Led, China) to remove the remaining cells and cellular debris. Then the filtered solution was transferred to a 40 mL sterile centrifuge tube (Merck-Millipore) and centrifuged at 10,000 × g for 30 min to remove cellular debris. The supematant was centrifugation use 40 mL sterile Ultra-Clea tube (Beckman Coulter, Brea, USA) and ultracentrifuged at 100,000 × g for 70 min at 4°C. Use a pipette to slowly remove the supernatant and add PBS, resuspended. Finally repeat the ultracentrifuged at 100,000 × g for 70 min at 4°C. The sediment at the bottom of the centrifuge tube is exosome. Measurement of the exosome concentration was performed using a microplate reader (ELX800, Bio-Rad, USA).

**Nanoparticle Tracking Analysis (NTA) of exosomes**

The size distribution of the exosomes was measured using Nanoparticle Tracking technology (Malvern Instruments, Malvern, UK), and was analysed using Nanoparticle Tracking Analysis software (Malvern).

**TEM analysis of exosomes**

The morphology of the exosomes was observed by transmission electron microscopy (TEM). Take 10 μL of the exosomal suspension drop on the nickel mesh, dry it at room temperature, drop it on the nickel mesh, dry at room temperature for 10 min, after glutaraldehyde fixation, negative stain with 3% phosphotungstic acid solution for 5 min, and dry at room temperature. dried and then observed using a Talos F200C TEM microscope (F200C, Thermo Fisher, USA).

**Exosome uptake by chondrocytes**

The separated exosomes were resuspended by PBS solution and ultra-centrifuged at 4°C for 70 min at 120,000×g. The exosomes were labeled according to the instruction of the PKH-26 (green) Fluorescent Cell Connection Kit (Millipore Sigma, Burlington, MA, USA). The precipitation of exosomes was washed with 100 ml medium and resuspended in 700 ml diluent C to prepare the exosome solution. The PKH-26 dye (1 ml) was diluted in 250 ml diluent C to solution. Next, 250 ml exosomes solution and 250 ml of PKH-26 solution were mixed in a 5 ml centrifuge tube. The samples were gently mixed for 4 min and added with 4.2 ml of 1% exosome-depleted bovine serum albumin to bind to excess PKH-26. The exosomes labeled by PKH-26 were centrifuged by the Optima TLX Ultra Centrifuge (Beckman Coulter, Brea, CA, USA) at 4°C for 70 min at 120,000× g. Next, the precipitation from the exosomes was washed with PBS solution 3 times. The chondrocytes cells were spread in 6-well slides at a density of 1 × 10^4^ cells/well in order to determine the relationship between exosomes and chondrocytes cells. After adding 5 μl of PKH-26 solution into the suspension, the mix was incubated at 37°C in 5% humidified CO_2_ and incubated with chondrocytes for 6 h. After washing three times in PBS to remove free exosomes, the chondrocytes were fixed with 4% paraformaldehyde for 15 min and stained with DAPI for 5 min. Finally, the images were photographed using a Leica DMI6000B fluorescence microscope (Leica Microsystems, Wetzlar, Germany).

1. **quantitative Real-time polymerase chain reaction (qRT-PCR)**

**The primer sequences of mRNA are shown below**

| **Gene** | **Forward Primer (5′-3')** | **Reverse Primer (5′-3')** |
| --- | --- | --- |
| RUNX2 | TATGAAAAACCAAGTAGCAAGGTTC | GTAATCTGACTCTGTCCTTGTGGAT |
| COL-Ⅱ | CAAGAACAGCATTGCCTATCTG | GATAACAGTCTTGCCCCACTTA |
| AGG | GAGGTGGTGGTGAAAGGTGT | GTGTGGATGGGGTACCTGAC |
| SOX9 | TCAACGGCTCCAGCAAGAAC | CTCCGCCTCCTCCACGAAGG |
| YAP | CGTCATGGGTGGCAGCAACTC | TCAGCCGCAGCCTCTCCTTC |
| CTGF | ATTCTGTGGAGTATGTACCGAC | GTCTCCGTACATCTTCCTGTAG |
| ANKRD1 | CCTGCGAGGCTGATCTCAATGC | GTCCGCACCGAAGTCATCAAG |
| GAPDH | TGGAATCCACTGGCGTCTTC | GGTTCACGCCCATCACAAAC |

1. **Isolation and characterization of articular chondrocytes**

Isolation and cultivation of rabbit articular chondrocytes (rbACs). Male rabbit (n=12; body weight 2.5±0.5 kg) provided by the Wuhan wan qian jia xing Biotech Co., Ltd (Lenience No. SCXK Hubei 2018-0011) were dissected following sacrifice and bilateral knee articular cartilage specimens were extracted and cut into 1 mm3 pieces. rbACs were isolated using a sequential proteinase and collagenase digestion technique. The cartilage sections were treated with PBS containing 1% penicillin and streptomycin (Gibco; Thermo Fisher Scientific, Inc., Waltham, MA, USA) for 3 times, then at 37˚C and 0.2% collagenase II (Gibco; Thermo Fisher Scientific, Inc.) overnight at 37˚C. Subsequently, the rbACs were resuspended and cultured in DMEM-F12 (1:1; Hyclone; GE Healthcare Life Sciences, Logan, UT, USA) supplemented with 10% fetal bovine serum (Gibco; Thermo Fisher Scientific, Inc.), containing 1% penicillin and streptomycin. The rbACs were expanded in monolayer culture in the medium in an incubator (Thermo Fisher Scientific, Inc.) at 37˚C under a humidified atmosphere containing 5% CO_2_, with an exchange of the medium every 3 days. When rbACs reached 90% confluence they were passaged at a ratio of 1:3 using 0.25% trypsin containing 0.05% EDTA. The rbACs of the third passage (P3) were seeded in 6-well plates at a density of 10^5^ cells/cm^2^ prior to treatment to avoid phenotype loss. Identify rbACs using toluidine blue staining, acridine orange fluorescent staining and type II collagen immunofluorescent staining.
